# A systematic review of changing malaria disease burden in sub-Saharan Africa since 2000: comparing model predictions and empirical observations

**DOI:** 10.1186/s12916-020-01559-0

**Published:** 2020-04-29

**Authors:** Alice Kamau, Polycarp Mogeni, Emelda A. Okiro, Robert W. Snow, Philip Bejon

**Affiliations:** 1 KEMRI-Wellcome Trust Research Programme, Kilifi, Kenya; 2 Centre for Tropical Medicine and Global Health, Nuffield Department of Clinical Medicine, University of Oxford, Oxford, UK

**Keywords:** Malaria Atlas Project, Incidence, Prevalence, Malaria, Systematic review, Africa, *Plasmodium falciparum*, Correlation

## Abstract

**Background:**

The most widely used measures of declining burden of malaria across sub-Saharan Africa are predictions from geospatial models. These models apply spatiotemporal autocorrelations and covariates to parasite prevalence data and then use a function of parasite prevalence to predict clinical malaria incidence. We attempted to assess whether trends in malaria cases, based on local surveillance, were similar to those captured by Malaria Atlas Project (MAP) incidence surfaces.

**Methods:**

We undertook a systematic review (PROSPERO International Prospective Register of Systematic Reviews; ID = CRD42019116834) to identify empirical data on clinical malaria in Africa since 2000, where reports covered at least 5 continuous years. The trends in empirical data were then compared with the trends of time-space matched clinical malaria incidence from MAP using the Spearman rank correlation. The correlations (*rho*) between changes in empirically observed and modelled estimates of clinical malaria were displayed by forest plots and examined by meta-regression.

**Results:**

Sixty-seven articles met our inclusion criteria representing 124 sites from 24 African countries. The single most important factor explaining the correlation between empirical observations and modelled predictions was the slope of empirically observed data over time (*rho* = − 0.989; 95% CI − 0.998, − 0.939; *p* < 0.001), i.e. steeper declines were associated with a stronger correlation between empirical observations and modelled predictions. Factors such as quality of study, reported measure of malaria and endemicity were only slightly predictive of such correlations.

**Conclusions:**

In many locations, both local surveillance data and modelled estimates showed declines in malaria burden and hence similar trends. However, there was a weak association between individual surveillance datasets and the modelled predictions where stalling in progress or resurgence of malaria burden was empirically observed. Surveillance data were patchy, indicating a need for improved surveillance to strengthen both empiric reporting and modelled predictions.

## Background

The burden of malaria has been reported to have declined across sub-Saharan Africa (SSA) [1–3]. However, uncertainty on the magnitude of this transition remains, and it is unclear to what extent the decline is spatially uniform across SSA [4–6]. Our current understanding of the declining burden of malaria across much of SSA has relied on modelled estimations based on historical epidemiological data, predictions in time and space based on sparse parasite prevalence data, and presumed impacts of interventions [1, 7, 8].

The traditional metric for classifying the quantity of malaria in a given location, or endemicity, has been parasite prevalence (referred to in literature as the “parasite rate (PR)”), which is derived from community-based surveys of infection [2, 9]. In current models of malaria disease burden in African, empirical data on 27,573 spatially and temporally unique PR observations are first modelled in time and space using a range of environmental, population and intervention covariates to provide approximately nine hundred thousand 5 × 5 km gridded surfaces of estimated malaria prevalence for every year between 2000 and 2015 [1]. These surfaces are then used in conjunction with limited historical epidemiological data on disease incidence, malaria-specific mortality and case-fatality rates [1, 8] to predict both malaria clinical incidence and mortality at a 5 × 5 km grid surface across SSA based on an estimated function of PR [1, 8]. The uncertainty in the predictions of parasite prevalence and disease burden was measured through out-of-bag sampling and reported as Bayesian credible intervals [1, 8]. The World Health Organization (WHO) has adopted these methods to estimate malaria burden in SSA countries where routine data is thought to be unreliable. During the estimations for 2017, the WHO [10] included adjusted and unadjusted empirical routine data for Botswana, Cape Verde, Comoros, Ethiopia, Namibia, Rwanda, Sao Tome and Principe, South Africa and Swaziland (Eswatini). In 2018, the list of countries where routine data was used expanded to include Eritrea, the Gambia, Madagascar, Mauritania, Mayotte, Senegal and Zimbabwe [11]. In 2019, the list was extended to include Djibouti [3].

Geostatistical and epidemiological models are rarely compared with empirical surveillance data. Here, the objective was to conduct a systematic review of published observations of temporal changes in malaria disease burden in SSA since 2000, updated from previous reviews [12–14], and compare this with the modelled predictions of changing clinical incidence of malaria over the same time period. In the Malaria Atlas Project (MAP) models, empirical data on PR observations are first modelled in time and space to provide estimated malaria prevalence. These surfaces of PR are then used to predict malaria clinical incidence at 5 × 5 km resolutions across Africa for each year between 2000 and 2015. We used these modelled predictions of changing clinical incidence of malaria to compare spatially matched clinical cases identified in our literature review over the same time period. While there are factors that might impact on malaria cases recorded by health facilities, we attempted to assess whether trends in malaria cases are in agreement with MAP incidence surfaces.

## Methods

### Search strategy and selection criteria

A systematic review and meta-analysis was conducted using PRISMA guidelines [15]. The protocol was registered in the PROSPERO International Prospective Register of Systematic Reviews (ID = CRD42019116834).

A literature search was performed in PubMed, MEDLINE, EMBASE, Web of Science and reference lists of publications between January 2000 and August 2018 on the test positivity rate/incidence of clinical malaria in SSA. A search strategy combining relevant terms and the names of the African countries was applied (Table 1). Studies considered included published papers in peer-reviewed journals, reports, book chapters and theses. We also manually screened citations of relevant articles to identify additional studies. In addition, we contacted authors of published hospital data to provide help with annual data not possible to extract directly from the published source.

**Table 1:** Search terms

(malaria OR plasmodium) AND	
(trend OR time series OR recession OR resurgence OR temporal OR decline OR increase OR change OR changing)	
AND	
(incidence OR prevalence) AND	
(Africa* OR Angola OR Benin OR Botswana OR Burkina Faso OR Burundi OR Cameroon OR Central African Republic OR Chad OR Congo* OR Cote d’Ivoire OR Equatorial Guinea OR Eritrea OR Ethiopia OR Gabon OR Gambia* OR Ghana OR Guinea* OR Kenya OR Liberia OR Madagascar OR Malawi OR Mali OR Mauritania OR Mauritius OR Mozambique OR Namibia OR Niger OR Nigeria OR Rwanda OR Senegal OR Sierra Leone OR Somalia OR Sudan OR Tanzania OR Togo OR Uganda OR Zambia OR Zimbabwe) AND	
“humans”[MeSH Terms] AND year=“2000-2018”	

For studies to be included in the review, they had to fulfill the following criteria: (i) articles reporting data from SSA; (ii) articles that included data on the following outcomes of interest: clinical malaria test positivity rate, malaria case period prevalence, incidence from facility-based surveillance, or community-based disease surveillance; and (iii) articles reporting continuous data for five or more years since 2000. We excluded studies that (i) reported data from countries where in 2017 the WHO used empirical routine data for morbidity estimation, and where most countries are near malaria elimination: Cape Verde, Comoros, Sao Tome and Principe, South Africa and Swaziland (Eswatini) [2, 10]; (ii) reported only repeat cross-sectional survey data on parasite prevalence or vector sporozoite rates in the community; (iii) reported modelled disease projections without empirical data; and (iv) reported only on verbal autopsy defined malaria mortality. For studies published in more than one report, the most comprehensive (years covered or availability of data for extraction) report was included.

The study selection process began by screening titles and abstracts retrieved from different electronic databases. We then reviewed the full text of eligible articles and compared all the retrieved articles with those included in the previous three reviews [12–14] to ensure no studies were missed.

### Data abstracted

Two authors (AK and PM) independently screened the articles for inclusion and extracted data on general information, i.e. first author name, year of publication, study location, country, source of data (hospital surveillance, cohort studies or clinic registers), sample size, age range, number of cases, test positive rate (defined as the number of positive malaria tests per 100 tests conducted), or incidence rate of malaria from tables, figures, text or summary data in the articles. Disagreements between reviewers were resolved through consensus. We used Gwet’s AC1 statistic to assess the inter-rater agreement for study inclusion [16].

### Critical appraisal

The quality of all included studies was assessed using the Joanna Briggs Institute Prevalence Critical Appraisal Tool [17]. Each study was assessed on 10 items; a score of 10% (yes) or 0% (no/unclear) was assigned and was summed across all items to generate an overall quality score that ranged from 0 to 100% (Additional file 1). Based on the overall score, we used two tertiles to split the studies into three groups. Studies were classified as having a high (< 34%), moderate (34–67%) or low (> 67%) risk of bias.

### Geographic information

Geo-referencing was undertaken through matching to previous health facility geo-coded master facility lists [18] and where not available using Google Earth or coordinates provided in the original publication. We assumed a representative study area for purposes of matching to modelled predictions of a radial distance of 30 km for each hospital [19], 10 km for large health centres and 5 km for dispensaries/clinics/health posts [20].

### Data analysis

Empirical data extracted from published reports were annualized, classified as hospital in-patient admissions, out-patient case burdens or community cohort incidence. An African region was assigned to each study based on the country of enrollment. The average endemicity at the start of the study’s surveillance period was defined using the MAP predicted *Plasmodium falciparum* parasite rate in children aged 2–10 years per site [1]. Study-level characteristics included the following: quality of study, geographic regions, source of data, reported measure of malaria, average starting parasite prevalence in children aged 2–10 years, sum of residuals, slope and sample size.

For each study, we time-space matched empirically recorded clinical incidence/test positivity rate of *P. falciparum* with modelled predictions of clinical malaria incidence developed by the MAP [1]. We extracted the mean clinical malaria incidence for all pixels in the specified raster that fell within the circular buffer for each geo-reference point for each year of the empirical surveillance data.

The correlation (*rho*) between changes in empirical clinical incidence/test positivity rate and modelled predictions of clinical malaria incidence was assessed using Spearman’s Rank correlation. Correlations were then classified as strong positive association (*rho* ≥ 0.6), moderate positive association (0.2 < *rho* < 0.6) or weak association (*rho* ≤ 0.2). A random effects meta-analysis was used to summarize the *rho* (Additional file 2); meta-analysis methods weight each study as a function of the between-study variance and within-study variance. We assessed the level of heterogeneity using Cochran’s *Q* statistic and *I*^2^. We used the forest plot to display the *rho* and the confidence intervals. Outliers were identified using the leave-one-out method, whereby we reran the meta-analysis iteratively removing studies. We used the funnel plots and Egger’s test to assess for publication bias [21].

To explore possible sources of heterogeneity, we performed meta-regression and sub-group analysis. In the sub-group analysis, the pooled correlation in each sub-group and within-group heterogeneity were obtained. In the meta-regression analysis, we examined the relationship between the study-level characteristics and the correlation of the two metrics using study as the unit of analysis [22]. Data analysis was performed using Stata, version 13 (Stata Corporation, College Station, TX) and R version 3.5.1 (R Core Team (2018), Vienna, Austria).

## Results

**Fig. 1:**
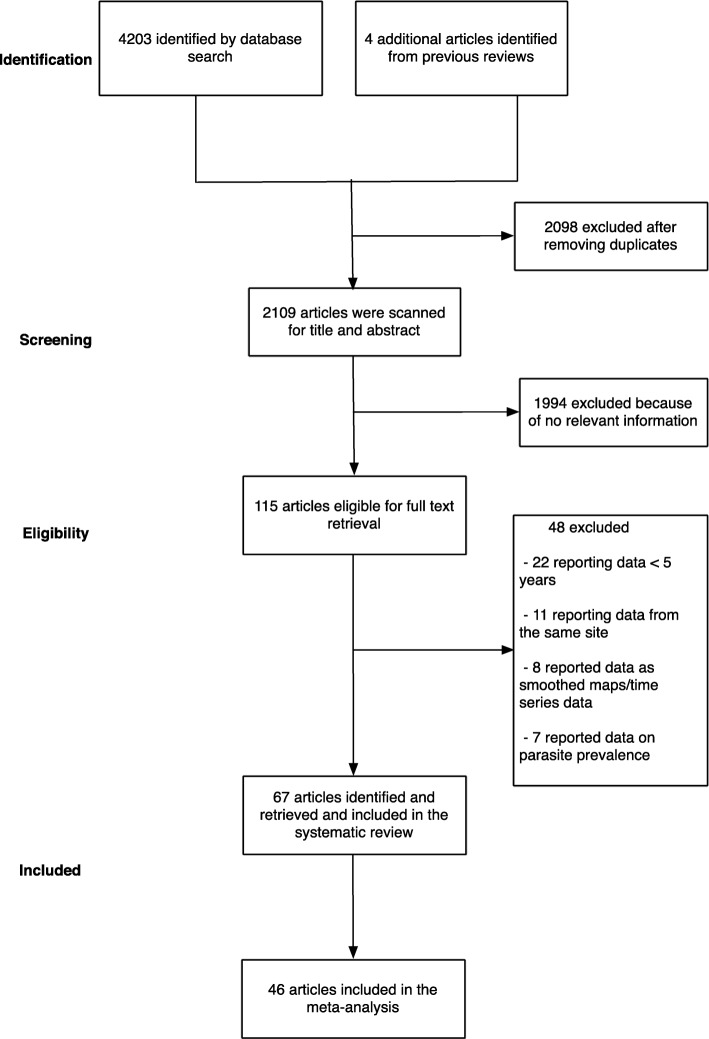
A summary flow of study selection process

The initial search yielded 4203 articles (Fig. 1). We then examined previous reviews [12–14] and found additional four articles that were relevant to our review which had been missed using our search terms [23–26]. We removed 2098 articles as they were duplicates between different electronic databases. After screening titles and abstracts of the remaining 2109 articles, we excluded 1994 articles because they did not fulfil the inclusion criteria. Following a full-text review of 115 articles, a further 48 articles were excluded because they either reported data from the same site, reported smoothed data, reported parasite prevalence or did not meet the complete 5-year restriction (Fig. 1). Finally, the study included data from 67 articles, where in some cases one publication reported data from more than one site. Three articles reported data from the same site but in different time periods, and/or from in-patient or out-patient records [27–29]. We obtained primary data, not possible to extract directly from the published source, for six publications related to hospital admissions at 24 sites across three countries (Malawi, Uganda and Kenya) (Additional file 3) [19, 30–34]. The level of agreement for study selection between the two authors was 96.4% (Gwet’s AC1 = 0.961), and disagreements were resolved after discussion.

**Fig. 2:**
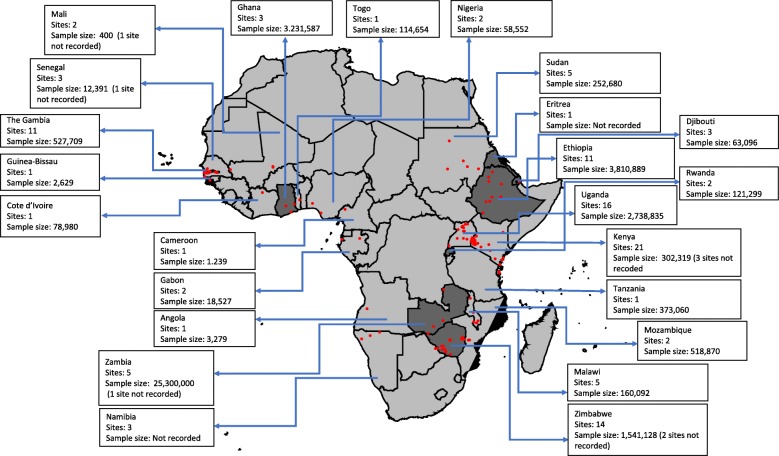
Assembled data included in the review by country, the number of sites and the sample size; dark grey indicates countries that reported national routine malaria case data, and the red dots indicate unique sites where data was identified

Nineteen (28%), twenty-seven (41%) and twenty-one (31%) articles were classified as having high, moderate and low risk of bias, respectively. The 67 articles resulted in a total of 124 geo-referenced locations where data was available for five or more years, covering 24 African countries since 2000 (Fig. 2). Data was obtained from the out-patient records (41%), in-patient records (30%), combined out-patient and in-patient records (27%), and cohort studies (2%) (Table 2).

**Table 2:** Characteristics of the included studies (124 geo-referenced locations from 67 articles)

Characteristics	Summary statistics
Geographical region, *n* (%)	
East Africa	45 (36.3%)
Southern Africa	29 (23.4%)
West Africa	26 (21.0%)
Horn of Africa	20 (16.1%)
Central Africa	4 (3.2%)
Quality of the study, *n* (%)	
High risk of bias	41 (32.5%)
Moderate risk of bias	52 (41.3%)
Low risk of bias	33 (26.2%)
Data source, *n* (%)	
In-patient	37 (29.8%)
In-patient and out-patient	34 (27.4%)
Out-patient	51 (41.1%)
Cohort studies	2 (1.6%)
Duration of reported data	
5 years	38 (30.6%)
6–9 years	42 (33.9%)
10–15 years	44 (35.5%)
Reported data spanning period	
2000–2005	5 (4.0%)
Post-2005	43 (34.7%)
Pre- and post-2005	76 (61.3%)
Average starting parasite prevalence in children aged 2–10 years	
< 10%	40 (32.3%)
10–50%	64 (51.6%)
> 50%	20 (16.1%)
Measure of malaria, *n* (%)	
Number of cases	40 (32.3%)
Test positive rate	62 (50.0%)
Incidence rate	22 (17.7%)
Data spatial level	
Country	10 (8.1%)
District	19 (15.3%)
Point	84 (67.7%)
Region	11 (8.9%)
Sample size, median (IQR)	18,389 (5889, 49,616)

Geographical region was classified as East Africa, Central Africa, Southern Africa and West Africa. Quality of study was classified as low, moderate and high risk of bias. Source of data was classified as in-patient and/or out-patient. Average starting parasite prevalence in children aged 2–10 years was classified as low, < 10%; moderate, 10–50%; or high, > 50%. Measure of malaria (incidence rate, test positivity rate or number of cases reported)

In the subsequent comparator analyses of modelled predictions of changing disease burden, 31/124 study sites from 21 publications were not included. The reasons for exclusion included the following: (i) 16 study sites in Ethiopia, Namibia and Rwanda classified as having reliable routine data to estimate malaria burden by WHO since the comparison would not be relevant in countries where surveillance data already feeds into the WHO report; (ii) 13 study sites in Djibouti, Eritrea, Ghana, Sudan, Zambia, Zanzibar and Zimbabwe that reported malaria case at national or regional level, the wide spatial extents of these data with likely inherent large variations in disease risk, precluding comparisons with modelled estimates; and (iii) two study sites (Dielmo, Senegal and Bandiagara, Mali) that reported community-cohort study data which we considered methodologically different from passive case detection (Additional file 3).

The remaining 93 sites from 46 publications included 28 (30%) sites that reported 5 years temporal data, 33 (36%) sites reported temporal data between 6 and 9 years and 32 (34%) sites reported data between 10 and 15 years. Three sites (3%) reported data between 2000 and 2005, 32 (34%) sites reported data post-2005 and 58 (63%) sites reported data spanning the period pre- and post-2005. The average starting parasite prevalence in children aged 2–10 years was < 10% in 19 sites, 10–50% in 53 sites and > 50% in 19 sites (Table 3). A sample of graphical presentation of extracted data, display of trend lines and the *rho* values were computed (Additional file 4).

**Table 3:** Sources of heterogeneity assessment based on meta-regression analyses (93 geo-referenced locations)

Factors	Summary statistics	Pooled correlation (*rho*)	95% CI	*p* value	Residual *I*^2^ (%)	Percentage change in *I*^2^ (%)
Geographical region, *n* (%)						
Eastern Africa	42 (45.2%)	0.43	0.18, 0.62	**0.01**	67.7	2.8
Southern Africa	23 (24.7%)	0.33	0.07, 0.54			
Western Africa	22 (23.7%)	0.73	0.53, 0.86			
Central Africa	4 (4.3%)	0.30	− 0.42, 0.79			
Horn of Africa	2 (2.1%)	0.84	0.60, 0.94			
Quality of the study, *n* (%)						
High risk of bias	28 (30.1%)	0.54	0.30, 0.71	**< 0.001**	63.2	9.3
Moderate risk of bias	41 (44.1%)	0.25	0.02, 0.46			
Low risk of bias	24 (25.8%)	0.78	0.63, 0.87			
Data source, *n* (%)						
In-patient	34 (36.6%)	0.36	0.07, 0.59	**0.01**	68.1	2.3
In-patient and out-patient	17 (18.3%)	0.38	0.03, 0.65			
Out-patient	42 (45.1%)	0.67	0.52, 0.78			
Measure of malaria, *n* (%)						
Number of cases reported	30 (32.3%)	0.26	− 0.01, 0.49	**0.001**	66.2	4.9
Test positive rate	47 (50.5%)	0.69	0.54, 0.79			
Incidence rate	16 (17.2%)	0.30	− 0.06, 0.59			
Average starting parasite prevalence in children aged 2–10 years						
< 10%	19 (20.4%)	0.33	− 0.08, 0.64	**0.002**	66.2	4.9
10–50%	55 (59.2%)	0.64	0.50, 0.75			
> 50%	19 (20.4%)	0.11	− 0.20, 0.41			
Sample size, median (IQR)	15,779 (3957, 35,981)	0.01	− 0.09, 0.11	0.83	71.8	− 3.1
Sum of residuals of empirical clinical incidence/test positivity rate, median (IQR)	0.94 (0.06, 2.05)	0.07	− 0.03, 0.17	0.07	69.3	0.5
Sum of residuals of MAP modelled clinical incidence, median (IQR)	0.12 (0.06, 0.30)	− 0.02	− 0.65, 0.62	0.24	69.8	− 0.3
Slope of empirical clinical incidence/test positivity rate, median (IQR)	− 0.003 (− 0.16, 0.001)	− 0.989	− 0.998, − 0.939	**< 0.001**	58.8	15.5
Slope of modelled MAP clinical incidence, median (IQR)	− 0.02 (− 0.03, 0.0001)	− 0.99995	− 1.00, − 0.997	**0.003**	67.5	3.1

Summary unadjusted *I*^2^ = 69.63%. Percentage change in *I*^2^ computed as (summary unadjusted *I*^2^ − residual *I*^2^)/summary unadjusted *I*^2^ × 100

Among the 93 sites included in the comparative analysis, 33% (31/93) showed evidence of a rise during the surveillance interval, and 67% (62/93) showed evidence of a decline. The matched modelled estimates predicted a rise in 23 sites and a decline in 70 sites. In 59 (63.4%) sites, the trends in both the empirical data and the modelled prediction were similar. However, 21 sites showed a rise in the empirical data while the modelled predictions showed a decline, and in 13 sites, the empirical data showed a decline while the modelled predictions showed a rise (Gwet’s AC1 = 0.378). In addition, 47% of the studies showed a strong positive association (*rho* ≥ 0.6), 15% (14/93) showed a moderate positive association (0.2 < *rho* < 0.6), and 38% (35/93) showed a weak association (*rho* ≤ 0.2) (Additional file 5). Eight sites, four in Kenya [19, 35], one in Mozambique [36], two in Uganda [28, 32] and one in Zimbabwe [37], showed strong negative correlations (*rho* ≤ − 0.6) (Additional file 5) indicating that the trends in the observed data versus the predicted data were markedly different. Four of the eight sites were in low malaria transmission areas and were classified as having a moderate risk of bias score. When we assessed for outliers using the leave-one-out method, there was no single study that was a substantial cause of heterogeneity (Additional file 6). Further, there was no evidence of publication bias (Egger’s test: *p* = 0.73 and Additional file 7). The summary meta-analysis of these studies showed a moderate positive association between empirically recorded changes in clinical incidence/test positivity rate and MAP modelled predictions of clinical malaria incidence (*rho* = 0.51; 95% CI 0.37, 0.63; *p* < 0.001; unadjusted *I*^2^ = 69.63%), indicating substantial heterogeneity.

When we explored possible sources of the observed heterogeneity by univariate analysis using meta-regression, we found that the slope of empirically recorded clinical incidence/test positivity rate (i.e. the rate of the empirically observed decline) was the single most important covariate, accounting for a 16% change in *I*^2^ (*rho* = − 0.989; 95% CI − 0.998, − 0.939; *p* < 0.001; residual *I*^2^ *=* 58.8%) (Table 3). The quality of data extracted, geographical region, reported measure of malaria, source of data, average starting parasite prevalence in children aged 2–10 years and slope of modelled clinical malaria incidence were additional sources of heterogeneity. However, the percentage change in *I*^2^ from these factors was minimal (which were responsible for percentage changes from 2% up to 9%, Table 3). Sample size and sum of residuals did not seem to explain the heterogeneity observed (Table 3).

**Fig. 3:**
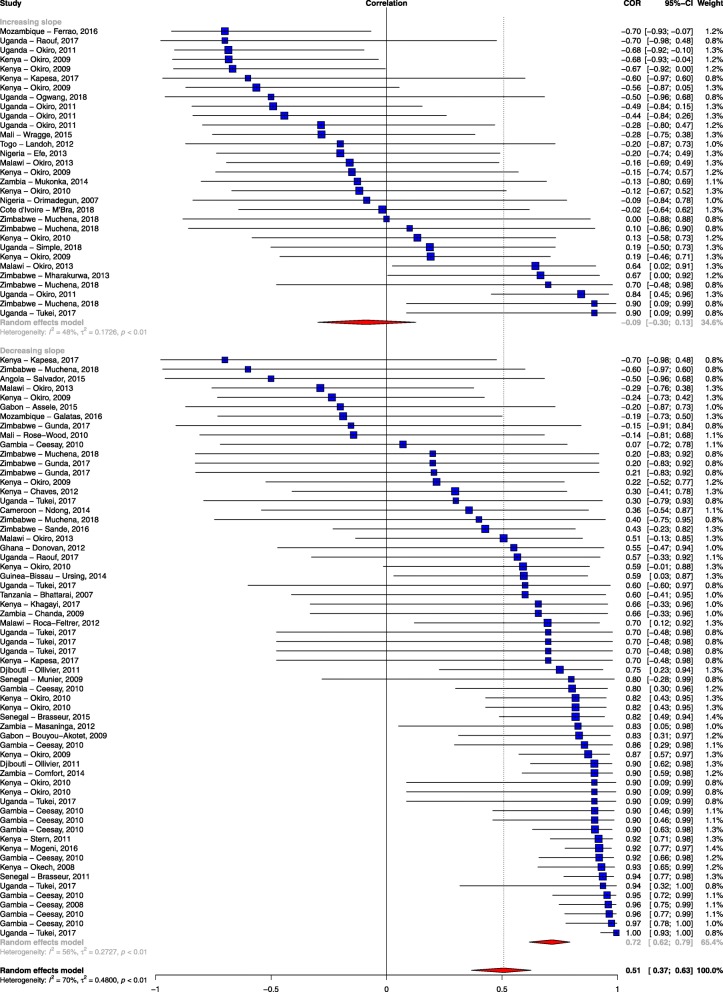
Forest plot of the correlation between empirical malaria cases and MAP clinical incidence stratified by the slope of empirical cases. Blue squares represent the correlation of each study; the error bars through the blue boxes are the uncertainty intervals; the red diamonds show the overall pooled correlation and in each sub-group; the horizontal tips of the red diamonds are the uncertainty level; weights are computed as the inverse of within and between variance; references are listed alphabetically in the Additional file material

The slope of clinical incidence/test positivity rate remained robust as a predictor of correlation even after excluding studies with a high risk of bias (*rho* = − 0.989; 95% CI − 0.998, − 0.923; *p* < 0.001) and after adjusting for the other study-level characteristics (*rho* = − 0.967; 95% CI − 0.997, − 0.689; *p* = 0.005; Additional file 8). As a sensitivity analysis, we examined stratifying the slope of empirically observed data as showing a decrease (< 0) or increase (≥ 0) and also found a significant association in the mean difference of the correlation between the observed data and the modelled prediction (*rho* = − 0.75; 95% CI − 0.86, − 0.60; *p* < 0.001; Fig. 3). We also examined the effect of the method of parasitological diagnosis and found a significant difference in the correlation between the observed data and modelled prediction when we stratified the analysis by the method of diagnosis (Additional file 9).

## Discussion

The demand for annual progress assessments of key malaria indicators has fueled the use of advanced mathematical models in order to estimate and predict malaria disease burdens and trends [1, 8, 38]. The application of these models has enabled the production of predictive maps of malaria risk [1, 8]. This review assesses whether trends in malaria cases from empiric surveillance are in agreement with the predictive model of malaria burden in SSA developed by MAP. We used empirically reported malaria data obtained from 93 health facility-based study sites from 46 published articles.

The empirical data obtain in this review included sites across the spectrum of malaria transmission intensity. The majority (60%) of sites were in moderate malaria transmission areas, 20% in low transmission areas and 20% in high transmission areas. There are previous reviews on the trends of malaria burden in Africa [12–14]. Our updated review is the first to make a comparison between empirical observations and modelled predictions.

Forty-seven percent of the study sites showed a strong positive association between empirical data and modelled predictions, while 38% of the studies showed a weak association. The single most important factor explaining this variation was the slope of empirically observed data over time (i.e. steep declines were associated with stronger correlations with the predicted estimates). If empirically recorded changes in malaria burden in a specific geographical area were on a downward trend, then the MAP modelled prediction was in agreement with this change (Fig. 3). Conversely, MAP modelled predictions showed a weak association with empiric surveillance data in sites that showed a stalling in progress or resurgence in empirically reported malaria burden. Although quality of study seemed to explain some of the variation, it was not a major cause of the heterogeneity observed when tested using meta-regression. One might expect studies classified as having a high risk of bias to correlate poorly with the modelled predictions, but this was not observed in our analysis (Table 3).

Although surveillance data on malaria cases recorded by health facilities were used, such data are not yet considered sufficiently reliable to track change in endemic countries in Africa [3]. This stems from varying clinical definitions of malaria between sites, changing case definition, changes in healthcare access and treatment-seeking behaviour, changes in diagnostic practice and reporting procedures or completeness within health systems which could have potentially affected the trends of malaria burden observed [3]. As a sensitivity analysis, we examined the effect of the method of diagnosis on the overall correlation. We found that the overall correlation among studies with parasitological confirmation was higher compared to studies with unclear methods of malaria diagnosis (Additional file 9). Nevertheless, even among those with documented parasitological confirmation, 41% (29/70) of the sites had *rho* < 0.5. While not all parasitologically diagnosed fevers are attributable to malaria [39], both coincidental infection and attributable fever may act as a valuable guide of malaria transmission. We could not evaluate the role of other factors on the impact of observed trends as most of the articles did not report on all the required information. However, our review was limited to data published in peer review journals with the assumption that data quality was examined during the peer review process.

Empirical reporting of malaria indicators should be a vital component of national control programs and now forms a central pillar of the Global Technical Strategy for malaria [40]. Improvements in routine data capture platforms [41], fever parasitological testing rates [42] and geospatial techniques to interpreting routine data [43, 44] have improved data availability in Africa [3]. In our review, assembled data from the published literature was patchy and was disproportionately from sites with research investment. Despite two decades of substantive investment in malaria control in Africa, we were able to identify only 67 published studies on the changing burden of malaria over this period. In many countries, routine data remain imperfect and are often unpublished, and hence, modelling will still be required unless further investments are made in surveillance capacity. Models are valuable in making predictions and generating hypotheses, and it is essential that commensurate investments are made in surveillance data to support and test these predictions. Another option is to develop sentinel hospitals to validate and use alongside current models of disease burden. While there are factors that might impact on the definition of a malaria case, temporal trends in hospitalized malaria cases may serve as a valuable barometer of community trends in disease burden and may have value more broadly than just in malaria [45].

This review had some limitations. Some articles did not report on all the required information. For example, only 31% of the articles included in this review had a low risk of bias due to the inherent methodological insufficiencies in the published articles. However, we acknowledge that the critical appraisal tool used in this review was based on a scale in which various components of the quality were scored and it is possible that it included fewer items that were critical in maintaining internal validity. In addition, some studies had 5 years’ worth of data which might have led to large uncertainty in the correlation. Secondly, there is limited published literature on trends of malaria risk in certain regions, particularly in central Africa. The studies reported in this review represent just 24 of the 45 countries reported to be endemic for malaria in WHO African region. Thirdly, although there was no evidence of publication bias, we must bear in mind the limitation of using published literature to validate the modelled prediction. For instance, bias might be introduced if publication of data that suggest malaria burden is worsening to encourage further investment in malaria control or data that reflects a decline to justify investment are favoured. More importantly, the use of incidence or number of cases could have led to over- or underestimation of malaria burden since passive surveillance can rarely be conducted with certainty regarding the population accessing care at any specific facility. Hence, variations in the population access to a local facility, behavioural changes or policy changes may lead to artefactual trends in malaria in the absence of actual variation in transmission.

## Conclusions

In many locations, both local surveillance data and modelled estimates showed declines in malaria burden and hence similar trends. However, there was spatial heterogeneity and some areas of Africa may have stable case numbers or upward trends. In these areas, there was a weak association between individual surveillance datasets and the modelled predictions. At broad regional scales, models may offer some guide on the overall trends in disease burden but have reduced predictive accuracy at local scales. The paucity of high quality, temporal clinical data in Africa must be redressed, to avoid a continued dependence on models or to help train future models. A public health priority should be high quality and temporally and spatially dense clinical data in Africa to empower national malaria control programs and to strengthen both empiric reporting and modelled predictions.

## Supplementary information

**Additional file 1.** Assessment of the quality of all studies included in the review based on Joanna Briggs Institute Prevalence Critical Appraisal Tool: Quality assessment tool for prevalence studies.

**Additional file 2.** Schematic outline of the steps taken in the data analysis.

**Additional file 3.** Summary characteristics of included studies, the first 31 study sites were not included in the meta-analysis for reasons outlined in the main manuscript.

**Additional file 4.** The graphical presentation of extracted data, display of trend lines and the *rho* values of the published studies and MAP model.

**Additional file 5.** Forest plot of ordered correlations between empirically recorded clinical incidence/test positivity rate of *P. falciparum* and MAP modelled predictions of clinical malaria disease incidence.

**Additional file 6.** Plots used to visualize the leave-one-out estimates, to identify outliers, or influential studies. We used a built-in function (Baujat Plot) using the package *metafor* in R statistical software where the outlying effect sizes were identified.

**Additional file 7.** Funnel plot with pseudo 95% confidence limits showing the Fisher’s Z transformed correlation coefficient (arctanh(r)) against the standard errors of arctanh(r).

**Additional file 8.** Sources of heterogeneity assessment based on multivariable meta-regression analyses.

**Additional file 9.** Forest plot of ordered correlation between empirically recorded clinical incidence/test positivity rate of *P. falciparum* and MAP modelled predictions of clinical malaria disease incidence stratified by method of diagnosis.

## Data Availability

A geo-coded repository of the data used for analysis is provided in Harvard Dataverse.

## Abbreviations

MAP: Malaria Atlas Project
*P. falciparum*: *Plasmodium falciparum*
PROSPERO: International Prospective Register of Systematic Reviews
*rho*: Correlation
SSA: Sub-Saharan Africa
PR: Parasite rate
WHO: World Health Organization
PRISMA: Preferred Reporting Items for Systematic Reviews and Meta-Analyses
