# Supplementary material for: A systematic review of changing malaria disease burden in sub-Saharan Africa since 2000: comparing model predictions and empirical observations
Source: BMC Med. 2020 Apr 29;18:94. doi: 10.1186/s12916-020-01559-0 (PMC7189714; doi:10.1186/s12916-020-01559-0)
Supplement: Supplementary file 1 — Additional file 1. Assessment of the quality of all studies included in the review based on Joanna Briggs Institute Prevalence Critical Appraisal Tool: Quality assessment tool for prevalence studies. [file 12916_2020_1559_MOESM1_ESM.docx]

**Additional file 1:** Assessment of the quality of all studies included in the review based on Joanna Briggs Institute Prevalence Critical Appraisal Tool: Quality assessment tool for prevalence studies

| 1st Author, year | Was the sample representative of the target population? | Were study participants recruited in an appropriate way? | Was the sample size adequate? | Were the study subjects and setting described in detail? | Is the data analysis conducted with sufficient coverage of the identified sample? | Were objective, standard criteria used for measurement of the condition? | Was the condition measured reliably? | Was there appropriate statistical analysis? | Are all important confounding factors/ subgroups/differences identified and accounted for? | Were subpopulations identified using objective criteria? | Quality score |
| --- | --- | --- | --- | --- | --- | --- | --- | --- | --- | --- | --- |
| **Alemu, A. 2011^1^** | U | U | U | Y | Y | Y | Y | Y | N | N | 50% |
| **Alemu, A. 2012^2^** | Y | Y | Y | Y | U | Y | U | Y | N | N | 60% |
| **Aregawi, M. 2014^3^** | Y | Y | Y | Y | N | Y | U | Y | N | N | 60% |
| **Aregawi, M. 2017^4^** | U | U | Y | Y | Y | Y | N | Y | N | N | 50% |
| **Aregawi, M. W. 2011^5^** | Y | Y | Y | Y | U | Y | N | Y | N | N | 60% |
| **Assele, V. 2015^6^** | Y | Y | Y | Y | U | Y | U | Y | N | N | 60% |
| **Bhattarai, A. 2007^7^** | U | U | Y | Y | Y | U | N | Y | N | N | 40% |
| **Bouyou-Akotet, M.  2009^8^** | Y | Y | Y | Y | U | Y | Y | Y | N | N | 70% |
| **Brasseur, P. 2015^9^** | Y | Y | Y | N | Y | Y | U | Y | N | N | 60% |
| **Brasseur, P., 2011^10^** | Y | Y | Y | Y | Y | Y | Y | Y | Y | Y | 100% |
| **Ceesay, S. J. 2008^11^** | Y | Y | Y | Y | U | Y | Y | Y | N | N | 70% |
| **Ceesay, S. J. 2010^12^** | Y | Y | Y | Y | Y | Y | N | Y | N | N | 70% |
| **Chanda, P. 2009^13^** | Y | Y | U | Y | U | Y | Y | Y | N | N | 60% |
| **Chaves, L. F., 2012^14^** | U | U | Y | N | U | U | U | Y | U | U | 20% |
| **Comfort, A. B. 2014^15^** | Y | Y | Y | Y | U | Y | U | Y | N | N | 60% |
| 1st Author, year | Was the sample representative of the target population? | Were study participants recruited in an appropriate way? | Was the sample size adequate? | Were the study subjects and setting described in detail? | Is the data analysis conducted with sufficient coverage of the identified sample? | Were objective, standard criteria used for measurement of the condition? | Was the condition measured reliably? | Was there appropriate statistical analysis? | Are all important confounding factors/ subgroups/differences identified and accounted for? | Were subpopulations identified using objective criteria? | Quality score |
| **Coulibaly, D. 2014^16^** | Y | Y | Y | Y | Y | Y | Y | Y | N | N | 80% |
| **Donovan, C 2012^17^** | Y | Y | Y | Y | N | N | N | Y | N | N | 50% |
| **Efe, SI 2013^18^** | U | U | U | Y | U | U | U | Y | N | N | 20% |
| **Eissa, F. H. 2017^19^** | U | U | U | Y | U | N | N | Y | N | N | 20% |
| **Ergete, S. 2018^20^** | Y | Y | Y | Y | Y | Y | U | Y | N | N | 70% |
| **Ferrao, J. L. 2016^21^** | Y | Y | Y | Y | Y | N | N | Y | N | N | 60% |
| **Galatas, B. 2016^22^** | Y | Y | Y | Y | Y | Y | Y | Y | N | N | 80% |
| **Gebretsadik, D. 2018^23^** | Y | Y | Y | Y | Y | Y | Y | Y | N | N | 80% |
| **Gunda, R. 2017^24^** | U | U | N | Y | Y | Y | N | Y | N | N | 40% |
| **Kamuliwo, M. 2013^25^** | U | U | U | Y | U | N | N | Y | N | N | 20% |
| **Kapesa, A. 2017^26^** | U | U | Y | U | U | Y | N | Y | N | N | 30% |
| **Khagayi, S. 2017^27^** | Y | Y | U | Y | U | Y | Y | Y | N | N | 60% |
| **Kigozi, Ruth 2012^28^** | Y | Y | Y | Y | Y | Y | U | Y | N | N | 70% |
| **Landoh, E. D. 2012^29^** | U | U | Y | Y | Y | N | N | Y | N | N | 40% |
| **M'Bra, R. K. 2018^30^** | N | N | Y | N | Y | N | N | Y | Y | Y | 50% |
| **Masaninga, F. 2012^31^** | U | U | Y | Y | U | N | N | Y | N | N | 30% |
| **Mba, Chuks J 2006^32^** | U | U | U | Y | U | N | N | Y | N | N | 20% |

| 1st Author, year | Was the sample representative of the target population? | Were study participants recruited in an appropriate way? | Was the sample size adequate? | Were the study subjects and setting described in detail? | Is the data analysis conducted with sufficient coverage of the identified sample? | Were objective, standard criteria used for measurement of the condition? | Was the condition measured reliably? | Was there appropriate statistical analysis? | Are all important confounding factors/ subgroups/differences identified and accounted for? | Were subpopulations identified using objective criteria? | Quality score |
| --- | --- | --- | --- | --- | --- | --- | --- | --- | --- | --- | --- |
| **Mharakurwa, S. 2013^33^** | U | U | Y | Y | N | N | N | Y | N | N | 30% |
| **Mogeni, P. 2016^34^** | Y | Y | Y | Y | Y | Y | Y | Y | Y | Y | 100% |
| **Muchena, G. 2018^35^** | U | U | N | N | U | N | N | U | N | N | 0% |
| **Mukonka, V. M. 2014^36^** | U | U | Y | N | U | N | N | Y | N | N | 20% |
| **Mukonka, V. M. 2015^37^** | U | U | Y | N | U | N | N | Y | N | N | 20% |
| **Munier, A. 2009^38^** | Y | Y | U | Y | U | N | N | Y | N | N | 40% |
| **Mutsigiri, F. 2017^39^** | U | U | Y | N | Y | Y | U | Y | N | N | 40% |
| **Ndong, I. C. 2014^40^** | N | N | N | Y | N | Y | N | Y | N | N | 30% |
| **Nyarango, P. M. 2006^41^** | U | U | U | Y | U | Y | N | Y | N | N | 30% |
| **Ogwang, R. 2018^42^** | Y | Y | Y | Y | Y | Y | U | Y | N | N | 70% |
| **Okech, B. A. 2008^43^** | Y | Y | Y | Y | U | Y | N | Y | N | N | 60% |
| **Okiro, E. A. 2009^44^** | Y | Y | U | Y | Y | N | N | Y | N | N | 50% |
| **Okiro, E. A. 2010^45^** | Y | Y | U | Y | Y | N | N | Y | N | N | 50% |
| **Okiro, E. A. 2011^46^** | Y | Y | Y | Y | Y | N | N | Y | N | N | 60% |
| **Okiro, E. A. 2013^47^** | Y | Y | Y | Y | U | N | N | Y | N | N | 50% |
| **Ollivier, L. 2011^48^** | Y | Y | Y | Y | Y | Y | U | Y | U | U | 70% |
| **Orimadegun, A. E. 2007^49^** | Y | Y | Y | Y | Y | Y | N | Y | N | N | 70% |

| 1st Author, year | Was the sample representative of the target population? | Were study participants recruited in an appropriate way? | Was the sample size adequate? | Were the study subjects and setting described in detail? | Is the data analysis conducted with sufficient coverage of the identified sample? | Were objective, standard criteria used for measurement of the condition? | Was the condition measured reliably? | Was there appropriate statistical analysis? | Are all important confounding factors/ subgroups/differences identified and accounted for? | Were subpopulations identified using objective criteria? | Quality score |
| --- | --- | --- | --- | --- | --- | --- | --- | --- | --- | --- | --- |
| **Otten, M. 2009^50^** | Y | Y | Y | Y | Y | N | N | Y | N | N | 60% |
| **Raouf, S. 2017^51^** | Y | Y | Y | Y | Y | Y | U | Y | N | U | 70% |
| **Roca-Feltrer, A. 2012^52^** | Y | Y | Y | Y | Y | Y | Y | Y | N | N | 80% |
| **Rose-Wood, Alyson 2010^53^** | Y | Y | U | Y | Y | N | N | Y | N | N | 50% |
| **Salvador, F. 2015^54^** | Y | Y | Y | Y | Y | Y | N | Y | N | N | 70% |
| **Sande, S. 2016^55^** | U | U | U | Y | U | U | U | U | N | U | 10% |
| **Sande, S. 2017^56^** | U | U | U | Y | U | Y | U | Y | N | U | 30% |
| **Sena, L. D. 2014^57^** | Y | Y | Y | Y | N | Y | U | Y | N | N | 60% |
| **Simple, O. 2018^58^** | U | U | Y | Y | U | N | N | Y | N | NA | 30% |
| **Smith Gueye, C 2014^59^** | U | U | U | Y | N | N | N | U | N | N | 10% |
| **Stern, D. I., 2011^60^** | U | U | Y | N | U | U | U | Y | U | U | 20% |
| **Tesfa, H. 2018^61^** | Y | Y | Y | Y | Y | Y | U | Y | N | N | 70% |
| **Trape, Jean-François 2014^62^** | Y | Y | Y | Y | Y | Y | Y | Y | Y | Y | 100% |
| **Tukei, B. B. 2017^63^** | U | U | Y | N | N | Y | U | Y | N | N | 30% |
| **Ursing, J. 2014^64^** | Y | Y | U | Y | Y | Y | Y | Y | N | N | 70% |
| **Wragge, S. E., 2015^65^** | Y | Y | Y | Y | N | Y | N | Y | U | U | 60% |
| **Yimer, F. 2015^66^** | Y | Y | Y | Y | Y | Y | Y | Y | N | N | 80% |
| **Yimer, M. 2017^67^** | Y | Y | Y | Y | Y | Y | Y | Y | N | N | 80% |

Each study was assessed on 10 items, a score of 10% (yes) or 0% (no/unclear) was assigned and was summed across all items to generate an overall quality score that ranged from 0% to 100%. Based on the overall score, we used two tertiles to split the studies into three groups. Studies were then classified as having a high (<34%), moderate (34%-67%) or low (>67%) risk of bias.
