# Supplementary material for: A systematic review of changing malaria disease burden in sub-Saharan Africa since 2000: comparing model predictions and empirical observations
Source: BMC Med. 2020 Apr 29;18:94. doi: 10.1186/s12916-020-01559-0 (PMC7189714; doi:10.1186/s12916-020-01559-0)
Supplement: Supplementary file 2 — Additional file 2. Schematic outline of the steps taken in the data analysis. [file 12916_2020_1559_MOESM2_ESM.docx]

**Additional file 2:** Schematic outline of the steps taken in the data analysis

Data assembled from the published studies reported changes in clinical cases of malaria for at least 5 continuous years. The assembled data was time-space matched with the modelled predictions of clinical malaria incidence derived from MAP. The magnitude of change between the empirical data and modelled prediction was assessed using the Spearman’s rank correlation. A random effects meta-analysis was used to summarize the correlations and their confidence intervals and displayed by forest plots. The meta-analysis methods weights each study as a function of the between-study variance and within-study variance. To explore possible sources of heterogeneity, a meta-regression and sub-group analysis were performed. In the sub-group analysis, studies were split into sub-groups according to the categorical covariate, the random-effect model was used to combine study effects within each sub-group. A pooled correlation in each sub-group and within-group heterogeneity was obtained. A meta-regression analysis was used to examine the relationship between the study-level characteristics and the correlation of the two metrics using study as the unit of analysis. Meta-regressions (i) allows larger studies to have more weight on the relationship than smaller studies, because studies are weighted by the precision of their effect estimate; and (ii) incorporates extra variability in the same way as in a random-effect meta-analysis i.e. allows for the residual heterogeneity among effects estimates not modelled by the study-level characteristics.
