# Supplementary material for: A systematic review of changing malaria disease burden in sub-Saharan Africa since 2000: comparing model predictions and empirical observations
Source: BMC Med. 2020 Apr 29;18:94. doi: 10.1186/s12916-020-01559-0 (PMC7189714; doi:10.1186/s12916-020-01559-0)
Supplement: Supplementary file 3 — Additional file 3. Summary characteristics of included studies, the first 31 study sites were not included in the meta-analysis for reasons outlined in the main manuscript. [file 12916_2020_1559_MOESM3_ESM.docx]

**Additional file 3:** Summary characteristics of included studies, the first 31 study sites were not included in the meta-analysis for reasons outlined in the main manuscript

| **Ref code** | **1st Author, year** | **Country** | **Site name** | **Data spatial level** | **Hospital in-patients/out-patients/morbidity incidence-cohort studies** | **Years** | **Sample size** | **Number of cases** |
| --- | --- | --- | --- | --- | --- | --- | --- | --- |
| 1 | Aregawi, M. 2014 | Ethiopia | 41 Hospitals, Ethiopia | Country | In-patient & Out-patient | 2001 - 2011 | 3,696,410 |  |
| 2 | Otten, M. 2009 | Ethiopia | 5 in-patient health facilities from Amhara, Tigray, and Afar states, Ethiopia | Country | In-patient | 2001 - 2007 | 13,106 | 4,920 |
| 3 | Otten, M. 2009 | Ethiopia | 8 facilities with out-patient data from Amhara, Tigray, Oromiya and SNNPR states, Ethiopia | Country | Out-patient | 2001 - 2007 | 252,840 | 29,600 |
| 4 | Alemu, A. 2011 | Ethiopia | Jimma town, Ethiopia | Point | In-patient & Out-patient | 2000 - 2009 |  | 19,798 |
| 5 | Alemu, A. 2012 | Ethiopia | Kola Diba Health Centre, Ethiopia | Point | Out-patient | 2002 - 2011 | 59,208 | 17,597 |
| 6 | Ergete, S. 2018 | Ethiopia | Hana and Keyafer Health Centres, Ethiopia | Point | Out-patient | 2008 - 2014 | 54,160 | 13,727 |
| 7 | Gebretsadik, D. 2018 | Ethiopia | Kombolcha Health Centre, Ethiopia | Point | Out-patient | 2009 - 2015 | 24,529 | 1,155 |
| 8 | Sena, L. D. 2014 | Ethiopia | Villages around a man-made lake, GGHD, within a distance of 10 km, Ethiopia | Point | Out-patient | 2004 - 2010 | 53,624 | 16,929 |
| 9 | Tesfa, H. 2018 | Ethiopia | Adi Arkay Health Centre, Ethiopia | Point | Out-patient | 2000 - 2013 | 18,782 | 4,467 |
| 10 | Yimer, F. 2015 | Ethiopia | Walga Health Centre, Ethiopia | Point | Out-patient | 2008 - 2012 | 34,060 | 5,889 |
| 11 | Yimer, M. 2017 | Ethiopia | Felegehiwot Referral Hospital, Ethiopia | Point | In-patient | 2010 - 2014 | 14,750 | 397 |
| 12 | Smith Gueye, C. 2014 | Namibia | Kunene Regions, Namibia | Region | In-patient & Out-patient | 2000 - 2011 | NR | NR |
| 13 | Smith Gueye, C. 2014 | Namibia | Ohangwena Regions, Namibia | Region | In-patient & Out-patient | 2000 - 2011 | NR | NR |
| 14 | Smith Gueye, C. 2014 | Namibia | Omusati Regions, Namibia | Region | In-patient & Out-patient | 2000 - 2011 | NR | NR |
| 15 | Otten, M. 2009 | Rwanda | 10/439 health centers out-patient cases, Rwanda | Country | In-patient | 2001 - 2007 | 366,711 | 71,683 |
| 16 | Otten, M. 2009 | Rwanda | 9/39 hospitals in-patient cases, Rwanda | Country | Out-patient | 2001 - 2007 | 93,829 | 49,616 |
| 17 | Ollivier, L. 2011 | Djibouti | Djibouti National Healthcare Insurance Program | Country | In-patient & Out-patient | 2001 - 2009 | 19,411 | 1,898 |
| 18 | Nyarango, P. M. 2006 | Eritrea | Eritrea | Country | In-patient & Out-patient | 2000 - 2004 | NR | NR |
| 19 | Aregawi, M. 2017 | Ghana | Ghana | Country | In-patient & Out-patient | 2005 - 2014 |  | 733,271 |
| 20 | Kamuliwo, M. 2013 | Zambia | Zambia | Country | In-patient & Out-patient | 2006 - 2014 |  | 53,052,500 |
| 21 | Sande, S. 2017 | Zimbabwe | Zimbabwe | Country | In-patient & Out-patient | 2003 - 2015 | NR | NR |
| **Ref code** | **1st Author, year** | **Country** | **Site name** | **Data spatial level** | **Hospital in-patients/out-patients/morbidity incidence-cohort studies** | **Years** | **Sample size** | **Number of cases** |
| 22 | Mba, Chuks J 2006 | Ghana | Volta Region, Ghana | Region | In-patient & Out-patient | 2000 - 2004 |  | 827,959 |
| 23 | Eissa, F. H. 2017 | Sudan | Gadaref State, Sudan | Region | In-patient & Out-patient | 2009 - 2013 |  | 26,567 |
| 24 | Eissa, F. H. 2017 | Sudan | Gezira State, Sudan | Region | In-patient & Out-patient | 2009 - 2013 |  | 108,006 |
| 25 | Eissa, F. H. 2017 | Sudan | Khartoum State, Sudan | Region | In-patient & Out-patient | 2009 - 2013 |  | 39,383 |
| 26 | Eissa, F. H. 2017 | Sudan | North Kordofan State, Sudan | Region | In-patient & Out-patient | 2009 - 2013 |  | 53,233 |
| 27 | Eissa, F. H. 2017 | Sudan | Northern State, Sudan | Region | In-patient & Out-patient | 2009 - 2013 |  | 25,491 |
| 28 | Aregawi, M. W. 2011 | Zanzibar (Tanzania) | 6 hospitals in Zanzibar, Tanzania | Region | Out-patient | 2000 - 2008 | 373,060 |  |
| 29 | Mutsigiri, F. 2017 | Zimbabwe | Manicaland Province, Zimbabwe | Region | In-patient & Out-patient | 2005 - 2014 |  | 947,462 |
| 30 | Trape, J. F. 2014 | Senegal | Dielmo village, Senegal | Point | Cohort | 2000 - 2012 | 5,445 |  |
| 31 | Coulibaly, D. 2014 | Mali | Bandiagara town, Mali | Point | Cohort | 2009 - 2013 | 400 |  |
| 32 | Salvador, F. 2015 | Angola | Hospital Nossa Senhora da Paz, Cubal, Angola | Point | In-patient & Out-patient | 2009 - 2013 | 23,106 | 3,279 |
| 33 | Ndong, I. C. 2014 | Cameroon | Mbakong Health Area, Mezam, Cameroon | Point | Out-patient | 2006 - 2012 | 4,158 | 1,239 |
| 34 | M'Bra, R. K. 2018 | Côte d'Ivoire | 4 Health Centres, Korhogo, Côte d'Ivoire | Point | Out-patient | 2004 - 2013 |  | 78,980 |
| 35 | Ollivier, L. 2011 | Djibouti | Bouffard French Military Hospital | Point | In-patient & Out-patient | 2002 - 2009 | 18,963 | 513 |
| 36 | Ollivier, L. 2011 | Djibouti | Peltier General Hospital | Point | In-patient & Out-patient | 2000 - 2009 | 24,722 | 1,436 |
| 37 | Assele, V. 2015 | Gabon | Urban Health Center and Regional Hospital of Makokou, Gabon | Point | Out-patient | 2006 - 2012 | 21,337 | 11,600 |
| 38 | Bouyou-Akotet, M.  2009 | Gabon | Centre Hospitalier de Libreville, Gabon | Point | In-patient & Out-patient | 2001 - 2008 | 26,603 | 6,927 |
| 39 | Ceesay, S. J. 2008 | Gambia | Brikama Health Centre, Gambia | Point | Out-patient | 2001 - 2009 | 85,600 |  |
| 40 | Ceesay, S. J. 2008 | Gambia | Fajara MRC outpatient Clinic, Gambia | Point | Out-patient | 2000 - 2009 | 161,631 |  |
| 41 | Ceesay, S. J. 2008 | Gambia | Keneba MRC Clinic, Gambia | Point | Out-patient | 2001 - 2009 | 7,686 |  |
| 42 | Ceesay, S. J. 2008 | Gambia | Sibanor Health Centre, Gambia | Point | Out-patient | 2001 - 2007 | 18,389 |  |
| 43 | Ceesay, S. J. 2010 | Gambia | Bansang Hospital, Gambia | Point | In-patient | 2003 - 2009 | 65,441 |  |
| 44 | Ceesay, S. J. 2010 | Gambia | Basse Hospital, Gambia | Point | In-patient | 2003 - 2009 | 14,070 |  |
| 45 | Ceesay, S. J. 2010 | Gambia | Sulayman Junkung Memorial Hospital, Bwiam, Gambia | Point | In-patient | 2004 - 2009 | 19,098 |  |
| 46 | Ceesay, S. J. 2010 | Gambia | Fajikunda Health Centre, Gambia | Point | Out-patient | 2003 - 2009 | 51,003 |  |
| 47 | Ceesay, S. J. 2010 | Gambia | Farafenni AFPRC Hospital, Gambia | Point | In-patient | 2003 - 2009 | 27,621 |  |
| **Ref code** | **1st Author, year** | **Country** | **Site name** | **Data spatial level** | **Hospital in-patients/out-patients/morbidity incidence-cohort studies** | **Years** | **Sample size** | **Number of cases** |
| 48 | Ceesay, S. J. 2010 | Gambia | Serekunda Health Centre, Gambia | Point | Out-patient | 2003 - 2009 | 61,786 |  |
| 49 | Ceesay, S. J. 2010 | Gambia | Soma Health Centre, Gambia | Point | Out-patient | 2003 - 2009 | 15,384 |  |
| 50 | Ursing, J. 2014 | Guinea-Bissau | Bandim Health Centre, Guinea-Bissau | Point | Out-patient | 2000 - 2012 |  | 2,629 |
| 51 | Chaves, L. F., 2012 | Kenya | Maseno Mission Hospital, Kenya | Point | In-patient | 2000 - 2009 |  | 3,957 |
| 52 | Kapesa, A. 2017 | Kenya | Iguhu, Kakamega County, Kenya | Point | Out-patient | 2011 - 2015 |  | 24,550 |
| 53 | Kapesa, A. 2017 | Kenya | Kombewa, Kisumu County, Kenya | Point | Out-patient | 2011 - 2015 |  | 28,550 |
| 54 | Kapesa, A. 2017 | Kenya | Marani, Kisii County, Kenya | Point | Out-patient | 2011 - 2015 |  | 39,250 |
| 55 | Mogeni, P. 2016 | Kenya | Kilifi County Hospital, Kenya | Point | In-patient | 2000 - 2014 |  | 15,145 |
| 56 | Okech, B. A. 2008 | Kenya | Kimbimbi sub-District Hospital, Mwea division, Kenya | Point | Out-patient | 2000 - 2007 | 46,842 | 9,392 |
| 57 | Okiro, E. A. 2009 | Kenya | Bondo District General Hospital, Kenya | Point | In-patient | 2000 - 2008 |  | 7,263 |
| 58 | Okiro, E. A. 2009 | Kenya | Bungoma District General Hospital, Kenya | Point | In-patient | 2000 - 2008 |  | 16,273 |
| 59 | Okiro, E. A. 2009 | Kenya | Busia District General Hospital, Kenya | Point | In-patient | 2000 - 2010 |  | 22,408 |
| 60 | Okiro, E. A. 2009 | Kenya | Homa Bay District General Hospital, Kenya | Point | In-patient | 2000 - 2010 |  | 16,174 |
| 61 | Okiro, E. A. 2009 | Kenya | Kitale District General Hospital, Kenya | Point | In-patient | 2000 - 2010 |  | 34,471 |
| 62 | Okiro, E. A. 2009 | Kenya | Makueni District General Hospital, Kenya | Point | In-patient | 2000 - 2008 |  | 2,814 |
| 63 | Okiro, E. A. 2009 | Kenya | Narok District General Hospital, Kenya | Point | In-patient | 2000 - 2008 |  | 4,884 |
| 64 | Okiro, E. A. 2009 | Kenya | Voi District General Hospital, Kenya | Point | In-patient | 2000 - 2010 |  | 6,984 |
| 65 | Okiro, E. A. 2009 | Kenya | Wajir District General Hospital, Kenya | Point | In-patient | 2000 - 2008 | 8,320 | 3,137 |
| 66 | Okiro, E. A. 2010 | Kenya | Kericho District General Hospital, Kenya | Point | In-patient | 2004 - 2008 | NR | NR |
| 67 | Okiro, E. A. 2010 | Kenya | Kisii District General Hospital, Kenya | Point | In-patient | 2000 - 2010 |  | 35,981 |
| 68 | Okiro, E. A. 2010 | Kenya | Kisumu District General Hospital, Kenya | Point | In-patient | 2004 - 2008 | NR | NR |
| 69 | Okiro, E. A. 2010 | Kenya | Malindi District General Hospital, Kenya | Point | In-patient | 2000 - 2010 |  | 7,437 |
| 70 | Okiro, E. A. 2010 | Kenya | Msambweni District General Hospital, Kenya | Point | In-patient | 2000 - 2010 |  | 6,332 |
| 71 | Okiro, E. A. 2010 | Kenya | Siaya District General Hospital, Kenya | Point | In-patient | 2000 - 2010 |  | 21,274 |
| 72 | Stern, D. I., 2011 | Kenya | Kericho Unilever Tea Kenya Ltd Hospital, Kenya | Point | In-patient | 2000 - 2010 |  | 3,439 |
| 73 | Okiro, E. A. 2013 | Malawi | Mwanza District General Hospital, Malawi | Point | In-patient | 2000 - 2010 | 34,461 | 18,293 |
| **Ref code** | **1st Author, year** | **Country** | **Site name** | **Data spatial level** | **Hospital in-patients/out-patients/morbidity incidence-cohort studies** | **Years** | **Sample size** | **Number of cases** |
| 74 | Okiro, E. A. 2013 | Malawi | Rumphi District General Hospital, Malawi | Point | In-patient | 2000 - 2010 | 25,914 | 12,636 |
| 75 | Okiro, E. A. 2013 | Malawi | Salima District General Hospital, Malawi | Point | In-patient | 2001 - 2010 | 40,771 | 18,746 |
| 76 | Okiro, E. A. 2013 | Malawi | Zomba District General Hospital, Malawi | Point | In-patient | 2000 - 2010 | 158,489 | 49,097 |
| 77 | Roca-Feltrer, A. 2012 | Malawi | Queen Elizabeth Central Hospital, Blantyre, Malawi | Point | Out-patient | 2001 - 2010 | 242,953 | 61,320 |
| 78 | Wragge, S. E., 2015 | Mali | Mine health department in the SEMOS mine clinics, Sadiola District, Mali | Point | Out-patient | 2004 - 2014 |  | 9,233 |
| 79 | Galatas, B. 2016 | Mozambique | Ilha Josina Machel Health Post, Mozambique | Point | Out-patient | 2004 - 2013 | 70,698 | 28,316 |
| 80 | Orimadegun, A. E. 2007 | Nigeria | University College Hospital, Ibadan, Nigeria | Point | In-patient | 2000 - 2005 | 16,031 | 1,806 |
| 81 | Brasseur, P. 2015 | Senegal | St Joseph dispensary of Mlomp, Senegal | Point | Out-patient | 2000 - 2012 | 19,089 | 6,946 |
| 82 | Brasseur, P., 2011 | Senegal | Mlomp village, Senegal | Point | Out-patient | 2000 - 2010 | 30,685 | 10,786 |
| 83 | Munier, A. 2009 | Senegal | 3 dispensaries in Niakhar Demographic Surveillance site, Senegal | Point | Out-patient | 2000 - 2004 | NR | NR |
| 84 | Kigozi, Ruth 2012 | Uganda | Aduku Health Centre, Uganda | Point | Out-patient | 2007 - 2015 | 164,596 |  |
| 85 | Ogwang, R. 2018 | Uganda | Kitgum Government Hospital2, Uganda | Point | In-patient | 2011 - 2015 | 58,496 | 21,766 |
| 86 | Okiro, E. A. 2011 | Uganda | Apac Hospital1, Uganda | Point | In-patient | 2000 - 2009 |  | 29,871 |
| 87 | Okiro, E. A. 2011 | Uganda | Jinja Hospital, Uganda | Point | In-patient | 2000 - 2009 |  | 35,189 |
| 88 | Okiro, E. A. 2011 | Uganda | Kambuga Hospital, Uganda | Point | In-patient | 2000 - 2010 |  | 20,300 |
| 89 | Okiro, E. A. 2011 | Uganda | Mubende Hospital, Uganda | Point | In-patient | 2001 - 2009 |  | 12,476 |
| 90 | Okiro, E. A. 2011 | Uganda | Tororo Hospital, Uganda | Point | In-patient | 2000 - 2009 |  | 43,887 |
| 91 | Raouf, S. 2017 | Uganda | Apac Hospital2, Uganda | Point | In-patient | 2011 - 2015 | 14,595 |  |
| 92 | Tukei, B. B. 2017 | Uganda | Awach Health Centre IV, Uganda | Point | Out-patient | 2007 - 2011 | 7,462 | 2,264 |
| 93 | Tukei, B. B. 2017 | Uganda | Gulu Independent hospital, Uganda | Point | Out-patient | 2007 - 2011 | 16,802 | 2,640 |
| 94 | Tukei, B. B. 2017 | Uganda | Gulu Regional Referral Hospital, Uganda | Point | Out-patient | 2007 - 2011 | 24,698 | 8,539 |
| 95 | Tukei, B. B. 2017 | Uganda | Kitgum Government Hospital1, Uganda | Point | Out-patient | 2007 - 2015 | 91,442 | 38,229 |
| 96 | Tukei, B. B. 2017 | Uganda | Lacor Hospital, Uganda | Point | Out-patient | 2007 - 2011 | 289,288 | 95,207 |
| 97 | Tukei, B. B. 2017 | Uganda | Lalogi Health Centre IV, Uganda | Point | Out-patient | 2007 - 2011 | 26,092 | 15,299 |
| 98 | Tukei, B. B. 2017 | Uganda | Military hospital, Uganda | Point | Out-patient | 2007 - 2011 | 11,347 | 2,981 |
| 99 | Tukei, B. B. 2017 | Uganda | Namokora Health Centre IV, Uganda | Point | Out-patient | 2007 - 2011 | 5,857 | 2,798 |
| **Ref code** | **1st Author, year** | **Country** | **Site name** | **Data spatial level** | **Hospital in-patients/out-patients/morbidity incidence-cohort studies** | **Years** | **Sample size** | **Number of cases** |
| 100 | Tukei, B. B. 2017 | Uganda | St Joseph's hospital, Uganda | Point | Out-patient | 2007 - 2011 | 75,915 | 30,097 |
| 101 | Chanda, P. 2009 | Zambia | Chongwe Rural Health Centre, Zambia | Point | Out-patient | 2003 - 2008 | NR | NR |
| 102 | Comfort, A. B. 2014 | Zambia | Macha Mission Hospital, Zambia | Point | Out-patient | 2000 - 2008 |  | 26,235 |
| 103 | Gunda, R. 2017 | Zimbabwe | Buvuma ward, Zimbabwe | Point | Out-patient | 2010 - 2014 |  | 74 |
| 104 | Gunda, R. 2017 | Zimbabwe | Ntalale ward, Zimbabwe | Point | Out-patient | 2010 - 2014 |  | 47 |
| 105 | Gunda, R. 2017 | Zimbabwe | Selonga ward, Zimbabwe | Point | Out-patient | 2010 - 2014 |  | 125 |
| 106 | Donovan, C 2012 | Ghana | 20 health facilities in Accra, Ghana | District | Out-patient | 2001 - 2006 | 4,308,469 | 1,670,357 |
| 107 | Khagayi, S. 2017 | Kenya | Siaya County, Kenya | District | Out-patient | 2007 - 2012 | NR | NR |
| 108 | Rose-Wood, Alyson 2010 | Mali | 7 Urban community health centres located in Mopti and Sévaré, Mali | District | Out-patient | 2000 - 2006 | NR | NR |
| 109 | Ferrao, J. L. 2016 | Mozambique | 6 public health centres and 1 Provincial hospital, Chimoio municipality, Mozambique | District | In-patient & Out-patient | 2006 - 2014 |  | 490,554 |
| 110 | Efe, SI 2013 | Nigeria | 5 health facilities from Warri metropolis , Nigeria | District | In-patient & Out-patient | 2000 - 2009 |  | 56,746 |
| 111 | Landoh, E. D. 2012 | Togo | Est Mono district, Togo | District | Out-patient | 2005 - 2010 | 222,338 | 114,654 |
| 112 | Simple, O. 2018 | Uganda | Gulu District, Uganda | District | Out-patient | 2006 - 2015 |  | 2,304,537 |
| 113 | Masaninga, F. 2012 | Zambia | Livingstone District, Zambia | District | Out-patient | 2004 - 2009 |  | 153,200 |
| 114 | Mukonka, V. M. 2014 | Zambia | Nchelenge District, Zambia | District | In-patient & Out-patient | 2006 - 2012 | 1,019,453 | 411,934 |
| 115 | Bhattarai, A., 2007 | Zanzibar (Tanzania) | 13 public health facilities North A district, Zanzibar, Tanzania | District | In-patient & Out-patient | 2000 - 2005 | 9,425 | 5,283 |
| 116 | Mharakurwa, S. 2013 | Zimbabwe | Mutasa District, Zimbabwe | District | In-patient & Out-patient | 2003 - 2011 | 1,971,621 | 587,120 |
| 117 | Muchena, G. 2018 | Zimbabwe | Beitbridge District | District | In-patient & Out-patient | 2011 - 2015 | NR | NR |
| 118 | Muchena, G. 2018 | Zimbabwe | Bulilima District | District | In-patient & Out-patient | 2011 - 2015 | NR | NR |
| 119 | Muchena, G. 2018 | Zimbabwe | Gwanda District | District | In-patient & Out-patient | 2011 - 2015 | NR | NR |
| 120 | Muchena, G. 2018 | Zimbabwe | Insiza District | District | In-patient & Out-patient | 2011 - 2015 | NR | NR |
| 121 | Muchena, G. 2018 | Zimbabwe | Mangwe District | District | In-patient & Out-patient | 2011 - 2015 | NR | NR |
| 122 | Muchena, G. 2018 | Zimbabwe | Matobo District | District | In-patient & Out-patient | 2011 - 2015 | NR | NR |
| 123 | Muchena, G. 2018 | Zimbabwe | Umzingwane District | District | In-patient & Out-patient | 2011 - 2015 | NR | NR |
| 124 | Sande, S. 2016 | Zimbabwe | Mutare District, Zimbabwe | District | In-patient & Out-patient | 2003 - 2013 | NR | NR |
