# Supplementary material for: A systematic review of changing malaria disease burden in sub-Saharan Africa since 2000: comparing model predictions and empirical observations
Source: BMC Med. 2020 Apr 29;18:94. doi: 10.1186/s12916-020-01559-0 (PMC7189714; doi:10.1186/s12916-020-01559-0)
Supplement: Supplementary file 4 — Additional file 4. The graphical presentation of extracted data, display of trend lines and the rho values of the published studies and MAP model. [file 12916_2020_1559_MOESM4_ESM.docx]

## **Additional file 4:** The graphical presentation of extracted data, display of trend lines and the *rho* values of the published studies and MAP model

Panel (a) represents MAP predicted clinical incidence of malaria (black line) and weekly malaria cases from Nossa Senhora da Paz hospital, Angola between the year 2009 -2013 (blue line).^54^ Panel (b) represents MAP predicted clinical incidence of malaria (black line) and laboratory malaria cases from Mbakong Health Area, Cameroon among patients of all ages between the years 2006 – 2012 (blue line).^40^ Panel (c) represents MAP predicted clinical incidence of malaria (black line) and clinical malaria cases from four health centres, Cote d’Ivoire among patients of all ages between the years 2004-2013 (blue line).^30^ Panel (d) represents MAP predicted clinical incidence of malaria (black line) and laboratory confirmed cases of malaria from Bouffard French Military hospital, Djibouti among patients of all ages between 2000 – 2009 (blue line).^48^

^^

Panel (a) represents MAP predicted clinical incidence of malaria (black line) and laboratory confirmed cases of malaria from Peltier General hospital, Djibouti among patients of all ages between 2000 – 2009 (blue line).^48^ Panel (b) represents MAP predicted clinical incidence of malaria (black line) and laboratory malaria cases from Makokou, Gabon among patients of all ages between the years 2006 – 2012 (blue line).^6^ Panel (c) represents MAP predicted clinical incidence of malaria (black line) and cases screened for malaria from Centre Hospitalier de Libreville, Gabon among patients aged < 11 years between the years 2001-2008 (blue line).^8^ Panel (d) represents MAP predicted clinical incidence of malaria (black line) and laboratory haematological malaria cases from Bansang hospital, The Gambia among children aged < 15 years between the years 2003 – 2009 (blue line).^11,12^

Panel (a) represents MAP predicted clinical incidence of malaria (black line) and laboratory haematological malaria cases from Basse hospital, The Gambia among children aged < 15 years between the years 2003 – 2009 (blue line).^11,12^ Panel (b) represents MAP predicted clinical incidence of malaria (black line) and laboratory haematological malaria cases from Brikama Health Centre, The Gambia among children aged < 15 years between the years 2001 – 2009 (blue line).^11,12^ Panel (c) represents MAP predicted clinical incidence of malaria (black line) and laboratory haematological malaria cases from Fajara MRC outpatient clinic, The Gambia among children aged < 15 years between the years 2000 – 2009 (blue line).^11,12^ Panel (d) represents MAP predicted clinical incidence of malaria (black line) and laboratory haematological malaria cases from Fajikunda Health Centre, The Gambia among children aged < 15 years between the years 2003 – 2009 (blue line).^11,12^

Panel (a) represents MAP predicted clinical incidence of malaria (black line) and laboratory haematological malaria cases from Farafenni AFPRC hospital, The Gambia among children aged < 15 years between the years 2003 – 2009 (blue line).^11,12^ Panel (b) represents MAP predicted clinical incidence of malaria (black line) and laboratory haematological malaria cases from Keneba MRC clinic, The Gambia among children aged < 15 years between the years 2001 – 2009 (blue line).^11,12^ Panel (c) represents MAP predicted clinical incidence of malaria (black line) and laboratory haematological malaria cases from Serekunda Health Centre, The Gambia among children aged < 15 years between the years 2003 – 2009 (blue line).^11,12^ Panel (d) represents MAP predicted clinical incidence of malaria (black line) and laboratory haematological malaria cases from Sibanor Health Centre, The Gambia among children aged < 15 years between the years 2001 – 2009 (blue line).^11,12^

Panel (a) represents MAP predicted clinical incidence of malaria (black line) and laboratory haematological malaria cases from Soma Healthh Centre, The Gambia among children aged < 15 years between the years 2003 – 2009 (blue line).^11,12^ Panel (b) represents MAP predicted clinical incidence of malaria (black line) and clinic reports on malaria cases from 20 health facilities in Accra, Ghana among patients of all ages between the years 2001 – 2006 (blue line).^17^ Panel (c) represents MAP predicted clinical incidence of malaria (black line) and malaria cases by microscopy from Bandim Health Centre, Guinea-Bissau among children aged < 15 years between the years 2000 – 2012 (blue line).^64^ Panel (d) represents MAP predicted clinical incidence of malaria (black line) and malaria cases from the outpatient records from Kimbimbi sub-District Hospital, Kenya among patients of all ages between the years 2000 – 2007 (blue line).^43^

Panel (a) represents MAP predicted clinical incidence of malaria (black line) and malaria cases from outpatient register from Iguhu, Kenya between the years 2011 – 2015 (blue line).^26^ Panel (b) represents MAP predicted clinical incidence of malaria (black line) and malaria cases from outpatient register from Kombewa, Kenya between the years 2011 – 2015 (blue line).^26^ Panel (c) represents MAP predicted clinical incidence of malaria (black line) and malaria cases from outpatient register from Marani, Kenya between the years 2011 – 2015 (blue line).^26^ Panel (d) represents MAP predicted clinical incidence of malaria (black line) and monthly malaria cases from Kericho Unilever Tea Ltd Hospital, Kenya between the years 2000 – 2010 (blue line).^60^

Panel (a) represents MAP predicted clinical incidence of malaria (black line) and malaria cases from clinic records from Maseno Mission Hospital, Kenya among patients of all ages between the years 2000 – 2009 (blue line).^14^ Panel (b) represents MAP predicted clinical incidence of malaria (black line) and in-patient malaria cases from Kilifi county hospital, Kenya among children aged 3 months – 13 years between the year 2000 -2014 (blue line).^34^ Panel (c) represents MAP predicted clinical incidence of malaria (black line) and malaria cases from inpatient register from Kericho District General Hospital, Kenya among children aged < 15 years between the years 2004 – 2008 (blue line).^45^ Panel (d) represents MAP predicted clinical incidence of malaria (black line) and malaria cases from inpatient register from Kisii District General Hospital, Kenya among children aged < 15 years between the years 2000 – 2010 (blue line).^45^

Panel (a) represents MAP predicted clinical incidence of malaria (black line) and malaria cases from inpatient register from Kisumu District General Hospital, Kenya among children aged < 15 years between the years 2004 – 2008 (blue line).^45^ Panel (b) represents MAP predicted clinical incidence of malaria (black line) and malaria cases from inpatient register from Malindi District General Hospital, Kenya among children aged < 15 years between the years 2000 – 2010 (blue line).^45^ Panel (c) represents MAP predicted clinical incidence of malaria (black line) and malaria cases from inpatient register from Msambweni District General Hospital, Kenya among children aged < 15 years between the years 2000 – 2010 (blue line).^45^ Panel (d) represents MAP predicted clinical incidence of malaria (black line) and malaria cases from inpatient register from Siaya District General Hospital, Kenya among children aged < 15 years between the years 2000 – 2010 (blue line).^45^

Panel (a) represents MAP predicted clinical incidence of malaria (black line) and malaria cases from 21 sentinel health facilities register from Siaya County, Kenya among children aged < 14 years between the years 2007 – 2012 (blue line).^27^ Panel (b) represents MAP predicted clinical incidence of malaria (black line) and malaria cases from inpatient register from Bungoma District General Hospital, Kenya among children aged < 15 years between the years 2000 – 2008 (blue line).^44^ Panel (c) represents MAP predicted clinical incidence of malaria (black line) and malaria cases from inpatient register from Bondo District General Hospital, Kenya among children aged < 15 years between the years 2000 – 2008 (blue line).^44^ Panel (d) represents MAP predicted clinical incidence of malaria (black line) and malaria cases from inpatient register from Busia District General Hospital, Kenya among children aged < 15 years between the years 2000 – 2010 (blue line).^45^

Panel (a) represents MAP predicted clinical incidence of malaria (black line) and malaria cases from inpatient register from Homa Bay District General Hospital, Kenya among children aged < 15 years between the years 2000 – 2010 (blue line).^45^ Panel (b) represents MAP predicted clinical incidence of malaria (black line) and malaria cases from inpatient register from Kitale District General Hospital, Kenya among children aged < 15 years between the years 2000 – 2010 (blue line).^45^ Panel (c) represents MAP predicted clinical incidence of malaria (black line) and malaria cases from inpatient register from Makueni District General Hospital, Kenya among children aged < 15 years between the years 2000 – 2008 (blue line).^44^ Panel (d) represents MAP predicted clinical incidence of malaria (black line) and malaria cases from inpatient register from Narok District General Hospital, Kenya among children aged < 15 years between the years 2000 – 2008 (blue line).^44^

Panel (a) represents MAP predicted clinical incidence of malaria (black line) and malaria cases from inpatient register from Voi District General Hospital, Kenya among children aged < 15 years between the years 2000 – 2010 (blue line).^45^ Panel (b) represents MAP predicted clinical incidence of malaria (black line) and malaria cases from inpatient register from Wajir District General Hospital, Kenya among children aged < 15 years between the years 2000 – 2008 (blue line).^44^ Panel (c) represents MAP predicted clinical incidence of malaria (black line) and daily outpatient records from seven health centres from Mopti and Sevare, Mali among children aged < 5 years between the years 2000 – 2006 (blue line).^53^ Panel (d) represents MAP predicted clinical incidence of malaria (black line) and malaria cases from clinic records from Mine health department in SEMOS, Mali among patients of all ages between the years 2004 – 2014 (blue line).^65^

Panel (a) represents MAP predicted clinical incidence of malaria (black line) and malaria cases from inpatient register from Mwanza District Hospital, Malawi among children aged < 5 years between the years 2000 – 2010 (blue line).^47^ Panel (b) represents MAP predicted clinical incidence of malaria (black line) and malaria cases from inpatient register from Rumphi District Hospital, Malawi among children aged < 5 years between the years 2000 – 2010 (blue line).^47^ Panel (c) represents MAP predicted clinical incidence of malaria (black line) and malaria cases from inpatient register from Salim District Hospital, Malawi among children aged < 5 years between the years 2001 – 2010 (blue line).^47^ Panel (d) represents MAP predicted clinical incidence of malaria (black line) and malaria cases from inpatient register from Zomba District Hospital, Malawi among children aged < 5 years between the years 2000 – 2010 (blue line).^47^

Panel (a) represents MAP predicted clinical incidence of malaria (black line) and malaria cases obtained from outpatient surveillance system from Ilha Josi, Mozambique among children aged < 15 years between the years 2004 – 2013 (blue line).^22^ Panel (b) represents MAP predicted clinical incidence of malaria (black line) and weekly malaria cases from district epidemiology bulletin from Chimoi municipality, Mozambique among patient of all ages between the years 2006 – 2014 (blue line).^21^ Panel (c) represents MAP predicted clinical incidence of malaria (black line) and malaria inpatient and outpatient cases from medical records from Warri metropolis, Nigeria between the years 2000 – 2009 (blue line).^18^ Panel (d) represents MAP predicted clinical incidence of malaria (black line) and malaria cases admitted to the emergency ward from University college hospital Ibadan, Nigeria among children aged < 15 years between the years 2000 – 2005 (blue line).^49^

Panel (a) represents MAP predicted clinical incidence of malaria (black line) and malaria cases obtained from registers from Niakhar Demographic surveillance site, Senegal among patients of all ages between the years 2000 – 2004 (blue line).^38^ Panel (b) represents MAP predicted clinical incidence of malaria (black line) and malaria cases obtained from clinic records from St Joseph dispensary Mlomp, Senegal among patient of all ages between the years 2000 – 2012 (blue line).^10^ Panel (c) represents MAP predicted clinical incidence of malaria (black line) and parasitological test results from clinic registers from Mlomp village, Senegal among patient of all ages between the years 2000 – 201o (blue line).^9^ Panel (d) represents MAP predicted clinical incidence of malaria (black line) and malaria cases from health information system from 17 health facilities in Est Mono district, Togo among patients of all ages between the years 2005 – 2010 (blue line).^29^

Panel (a) represents MAP predicted clinical incidence of malaria (black line) and malaria cases obtained from sentinel surveillance system from Aduku health centre, Uganda among patients of all ages between the years 2007 – 2015 (blue line).^28^ Panel (b) represents MAP predicted clinical incidence of malaria (black line) and malaria cases from inpatient register from Jinja Hospital, Uganda among children aged < 15 years between the years 2000 – 2009 (blue line).^46^ Panel (c) represents MAP predicted clinical incidence of malaria (black line) and malaria cases from inpatient register from Kambuga Hospital, Uganda among children aged < 15 years between the years 2000 – 2010 (blue line).^46^ Panel (d) represents MAP predicted clinical incidence of malaria (black line) and malaria cases from inpatient register from Mubende Hospital, Uganda among children aged < 15 years between the years 2001 – 2009 (blue line).^46^

^^

Panel (a) represents MAP predicted clinical incidence of malaria (black line) and malaria cases from inpatient register from Tororo Hospital, Uganda among children aged < 15 years between the years 2000 – 2009 (blue line).^46^ Panel (b) represents MAP predicted clinical incidence of malaria (black line) and malaria cases from inpatient register from Apac Hospital, Uganda among children aged < 15 years between the years 2000 – 2009 (blue line).^46^ Panel (c) represents MAP predicted clinical incidence of malaria (black line) and malaria cases from integrated health management information system from Awach Health Centre IV, Uganda among patients of all ages between the years 2007 – 2011 (blue line).^63^ Panel (d) represents MAP predicted clinical incidence of malaria (black line) and malaria cases from integrated health management information system from Gulu Independent Hospital, Uganda among patients of all ages between the years 2007 – 2011 (blue line).^63^

Panel (a) represents MAP predicted clinical incidence of malaria (black line) and malaria cases from integrated health management information system from Gulu Regional Referral Hospital, Uganda among patients of all ages between the years 2007 – 2011 (blue line).^63^ Panel (b) represents MAP predicted clinical incidence of malaria (black line) and malaria cases from integrated health management information system from Kitgum Government Hospital, Uganda among patients of all ages between the years 2007 – 2011 (blue line).^63^ Panel (c) represents MAP predicted clinical incidence of malaria (black line) and record-based study of inpatients cases from Kitgum Government Hospital, Uganda among patients of all ages between the years 2011 – 2015 (blue line).^42^ Panel (d) represents MAP predicted clinical incidence of malaria (black line) and malaria cases from integrated health management information system from Lacor Hospital, Uganda among patients of all ages between the years 2007 – 2011 (blue line).^63^

Panel (a) represents MAP predicted clinical incidence of malaria (black line) and malaria cases from integrated health management information system from Lalogi Health Centre IV, Uganda among patients of all ages between the years 2007 – 2011 (blue line).^63^ Panel (b) represents MAP predicted clinical incidence of malaria (black line) and malaria cases from integrated health management information system from Military Hospital, Uganda among patients of all ages between the years 2007 – 2011 (blue line).^63^ Panel (c) represents MAP predicted clinical incidence of malaria (black line) and malaria cases from integrated health management information system from Namokora Health Centre IV, Uganda among patients of all ages between the years 2007 – 2011 (blue line).^63^ Panel (d) represents MAP predicted clinical incidence of malaria (black line) and malaria cases from integrated health management information system from St Joseph’s Hospital, Uganda among patients of all ages between the years 2007 – 2011 (blue line).^63^

Panel (a) represents MAP predicted clinical incidence of malaria (black line) and malaria cases extracted from health management information system from Gulu District, Uganda among patients of all ages between the years 2006 – 2015 (blue line).^58^ Panel (b) represents MAP predicted clinical incidence of malaria (black line) and malaria cases from inpatient register from Apac Hospital, Uganda among children aged < 14 years between the years 2011 – 2015 (blue line).^51^ Panel (c) represents MAP predicted clinical incidence of malaria (black line) and malaria cases from integrated health management information system from Zanzibar- North A District, Tanzania among children aged < 5 years between the years 2000 – 2005 (blue line).^7^

Panel (a) represents MAP predicted clinical incidence of malaria (black line) and malaria cases from DHIS data collected from 11 health facilities from Nchelenge District, Zambia among patients of all ages between the years 2006 – 2012 (blue line).^36^ Panel (b) represents MAP predicted clinical incidence of malaria (black line) and malaria cases aggregated from hospital forms from Macha Mission Hospital, Zambia among children aged < 5 years between the years 2000 – 2008 (blue line).^15^ Panel (c) represents MAP predicted clinical incidence of malaria (black line) and malaria cases derived from clinic records from Livingstone District, Zambia between the years 2004 – 2009 (blue line).^31^ Panel (d) represents MAP predicted clinical incidence of malaria (black line) and parasitemia data from Chongwe Rural Health Centre, Zambia among children aged < 5 years between the years 2003 – 2008 (blue line).^13^

Panel (a) represents MAP predicted clinical incidence of malaria (black line) and malaria cases from DHIS 3 from Mutare District, Zimbabwe among patients of all ages between the years 2003 – 2013 (blue line).^55^ Panel (b) represents MAP predicted clinical incidence of malaria (black line) and malaria cases based on malaria surveillance system from Beitbridge District, Zimbabwe among patients of all ages between the years 2011– 2015 (blue line).^35^ Panel (c) represents MAP predicted clinical incidence of malaria (black line) and malaria cases based on malaria surveillance system from Bulilima District, Zimbabwe among patients of all ages between the years 2011– 2015 (blue line).^35^ Panel (d) represents MAP predicted clinical incidence of malaria (black line) and malaria cases based on malaria surveillance system from Gwanda District, Zimbabwe among patients of all ages between the years 2011– 2015 (blue line).^35^

Panel (a) represents MAP predicted clinical incidence of malaria (black line) and malaria cases based on malaria surveillance system from Insiza District, Zimbabwe among patients of all ages between the years 2011– 2015 (blue line).^35^ Panel (b) represents MAP predicted clinical incidence of malaria (black line) and malaria cases based on malaria surveillance system from Mangwe District, Zimbabwe among patients of all ages between the years 2011– 2015 (blue line).^35^ Panel (c) represents MAP predicted clinical incidence of malaria (black line) and malaria cases based on malaria surveillance system from Matobo District, Zimbabwe among patients of all ages between the years 2011– 2015 (blue line).^35^ Panel (d) represents MAP predicted clinical incidence of malaria (black line) and malaria cases based on malaria surveillance system from Umzingwane District, Zimbabwe among patients of all ages between the years 2011– 2015 (blue line).^35^

Panel (a) represents MAP predicted clinical incidence of malaria (black line) and malaria cases from DHIS and health facilities from Buvuma ward, Zimbabwe among patients of all ages between the years 2010– 2014 (blue line).^24^ Panel (b) represents MAP predicted clinical incidence of malaria (black line) and malaria cases from DHIS and health facilities from Ntalale ward, Zimbabwe among patients of all ages between the years 2010– 2014 (blue line).^24^ Panel (c) represents MAP predicted clinical incidence of malaria (black line) and malaria cases from DHIS and health facilities from Selonga ward, Zimbabwe among patients of all ages between the years 2010– 2014 (blue line).^24^ Panel (d) represents MAP predicted clinical incidence of malaria (black line) and malaria cases obtained from health facilities that offer inpatient services from Mutasa District, Zimbabwe among patients aged ≥ 5 years between the years 2003– 2011 (blue line).^33^
