# Supplementary material for: A systematic review of changing malaria disease burden in sub-Saharan Africa since 2000: comparing model predictions and empirical observations
Source: BMC Med. 2020 Apr 29;18:94. doi: 10.1186/s12916-020-01559-0 (PMC7189714; doi:10.1186/s12916-020-01559-0)
Supplement: Supplementary file 6 — Additional file 6. Plots used to visualize the leave-one-out estimates, to identify outliers, or influential studies. We used a built-in function (Baujat Plot) using the package metafor in R statistical software where the outlying effect sizes were identified. [file 12916_2020_1559_MOESM6_ESM.docx]

**Additional file 6**: Plots used to visualize the leave-one-out estimates, to identify outliers, or influential studies. We used a built-in function (Baujat Plot) using the package *metafor* in R statistical software where the outlying effect sizes were identified
