# Supplementary material for: A systematic review of changing malaria disease burden in sub-Saharan Africa since 2000: comparing model predictions and empirical observations
Source: BMC Med. 2020 Apr 29;18:94. doi: 10.1186/s12916-020-01559-0 (PMC7189714; doi:10.1186/s12916-020-01559-0)
Supplement: Supplementary file 7 — Additional file 7. Funnel plot with pseudo 95% confidence limits showing the Fisher’s Z transformed correlation coefficient (arctanh(r)) against the standard errors of arctanh(r). [file 12916_2020_1559_MOESM7_ESM.docx]

**Additional file 7**: Funnel plot with pseudo 95% confidence limits showing the Fisher’s Z transformed correlation coefficient (arctanh(r)) against the standard errors of arctanh(r)
