# Supplementary material for: A systematic review of changing malaria disease burden in sub-Saharan Africa since 2000: comparing model predictions and empirical observations
Source: BMC Med. 2020 Apr 29;18:94. doi: 10.1186/s12916-020-01559-0 (PMC7189714; doi:10.1186/s12916-020-01559-0)
Supplement: Supplementary file 8 — Additional file 8. Sources of heterogeneity assessment based on multivariable meta-regression analyses. [file 12916_2020_1559_MOESM8_ESM.docx]

# **Additional file 8:** Sources of heterogeneity assessment based on multivariable meta-regression analyses

| Factors | Meta-regression coefficient (Fisher’s Z transformed correlation coefficient) | [95% Cl] | P value | Holm-Bonferroni corrected P value |
| --- | --- | --- | --- | --- |
| **Geographical Region, n(%)** |  |  |  |  |
| Southern Africa | 0·19 | -0·37, 0·75 | 0·05 | 0·33 |
| Western Africa | 0·22 | -0·31, 0·75 |  |  |
| Central Africa | -0·43 | -1·35, 0·50 |  |  |
| Horn of Africa | 0·93 | -0·28, 2.14 |  |  |
| **Quality of the study, n(%)** |  |  |  |  |
| Moderate risk of bias | -0·48 | -0·95, -0·02 | 0·04 | 0·33 |
| Low risk of bias | -0·39 | -1.00, 0·22 |  |  |
| **Data source, n(%)** |  |  |  |  |
| In-patient and out-patient | -0·21 | -0·98, 0·56 | 0·09 | 0·46 |
| Out-patient | 0·21 | -0·22, 0·65 |  |  |
| **Measure of malaria, n(%)** |  |  |  |  |
| Test positive rate | 0·22 | -0·27, 0·70 | 0·19 | 0·58 |
| Incidence rate | -0·09 | -1·13, 0·94 |  |  |
| **Average starting endemicity** |  |  |  |  |
| 10-50% | 0·14 | -0·41, 0·68 | 0·04 | 0·33 |
| > 50% | -0·30 | -0·94, 0·34 |  |  |
| **Sample size, median (IQR)** | -0·04 | -0·15, 0·08 | 0·55 | 0·58 |
| **Sum of residuals of empirical clinical incidence/test positivity rate, median (IQR)** | -0·03 | -0·15, 0·09 | 0·27 | 0·58 |
| **Sum of residuals of MAP modelled clinical incidence, median (IQR)** | 0·49 | -0·26, 1·24 | 0·11 | 0·46 |
| **Slope of empirical clinical incidence/test positivity rate, median (IQR)** | -2·04 | -3·23, -0·85 | **<0·001** | **0·005** |
| **Slope of MAP modelled clinical incidence, median (IQR)** | -9·37 | -18·11, -0·64 | 0·02 | 0·18 |

**Geographical Region** (Eastern Africa vs Southern Africa/Western Africa/Central Africa/Horn of Africa)**; Quality of the study** (High risk of bias vs Moderate risk of bias/Low risk of bias); **Data source** (In-patient vs In-patient and out-patient/Out-patient); **Measure of malaria (**number of cases reported/test positive rate/incidence rate); **Average starting endemicity (**low: <10% vs moderate: 10 - 50%/high: >50%)
