# Supplementary material for: A systematic review of changing malaria disease burden in sub-Saharan Africa since 2000: comparing model predictions and empirical observations
Source: BMC Med. 2020 Apr 29;18:94. doi: 10.1186/s12916-020-01559-0 (PMC7189714; doi:10.1186/s12916-020-01559-0)
Supplement: Supplementary file 9 — Additional file 9. Forest plot of ordered correlation between empirically recorded clinical incidence/test positivity rate of P. falciparum and MAP modelled predictions of clinical malaria disease incidence stratified by method of diagnosis. [file 12916_2020_1559_MOESM9_ESM.docx]

**Additional file 9:** Forest plot of ordered correlation between empirically recorded clinical incidence/test positivity rate of P. falciparum and MAP modelled predictions of clinical malaria disease incidence stratified by method of diagnosis.

Blue squares represents the correlation of each study; the error bars through the blue boxes are the uncertainty intervals; the red diamonds shows the overall pooled correlation and in each subgroup; the horizontal tips of the red diamonds are the uncertainty level; weights are computed as the inverse of within and between variance; references are listed alphabetically in the *Additional file* material.
